# T-13 and T-26, the novel taxanes with improved oral bioavailability in rats

**DOI:** 10.1038/s41598-020-60184-2

**Published:** 2020-02-21

**Authors:** Yun-Rong Jing, Wei Zhou, Xiang-Yang Wang

**Affiliations:** 1 Department of Life Sciences and Technology, Mudanjiang Normal University, No.191, Wenhua Street, Aimin District, Mudanjiang, 157011 Heilongjiang PR China; 2 Chem-Pharm R&D Institute, Tianjin Tasly Group Co., Ltd, Tasly TCM Garden, No.2, Pujihe East Road, Beichen District, Tianjin PR China

**Keywords:** Pharmacokinetics, Drug discovery and development

## Abstract

In an attempt to improve the oral bioavailability of taxanes, a series of new analogues were synthesized and tested in a panel of human tumor cell lines and cellular permeability assays. Compounds T-13 and T-26 showed potent cytotoxicity and exhibited the highest permeability, so they were selected for pharmacokinetic studies. Here, pharmacokinetics of T-13 and T-26 were studied after intravenous injection (5 mg/kg) and oral administration (60 mg/kg) in male Sprague-Dawley (S.D.) rats, respectively. Plasma concentrations were characterized using liquid chromatography-tandem mass spectrometry (LC-MS/MS). The oral bioavailability of T-13 and T-26 was determined to be 10.71% and 65.79%, respectively. Compounds T-13 and T-26 were both poor substrates of P-glycoprotein (P-gp), and they had a much higher bioavailability than paclitaxel, especially T-26. T-26 with good oral bioavailability represented a potential candidate for potent antitumor activity given oral administration.

## Introduction

Paclitaxel(PTX) has poor aqueous solubility and its intestinal uptake is severely hampered by the ATP-binding cassette (ABC) drug efflux transporter P-glycoprotein (P-gp)^1–6^, which severely limit its oral bioavailability^7,8^. Therefore, PTX requires to be co-injected with the detergent Cremophor EL, and this detergent frequently causes untoward hypersensitivity reactions, which has created significant problems in developing suitable pharmaceutical formulations suitable for chemotherapy^9–11^.

**Figure 1:**
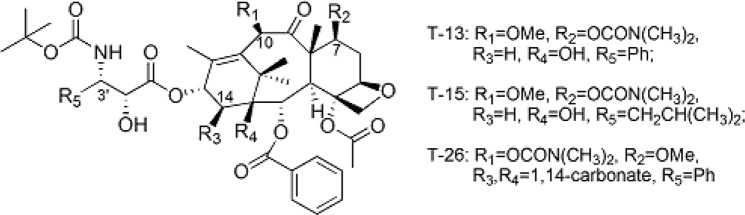
Taxanes bearing modifications at the key positions of C-7, C-10, C-14 and C-3′.

In an attempt to increase the solubility of taxoids and develop more safe clinical formulations, we previously synthesized a series of novel taxol analogs by modifying the positions of C7, C10, C14 and C3′, which have improved water solubility and oral bioavailability. Among these analogs, T-13, T-15 and T-26 (Fig. 1) exhibited the highest permeability in Caco-2 monolayer cells, with the efflux ratios of less than 2.0^12^. Three taxol analogs demonstrated the potent cytotoxic activities similar to paclitaxel in MCF-7 cancer cell lines. In order to further evaluate the substrate activity of novel taxol analogs with P-gp and whether they are P-gp inhibitors, T-13 and T-26 were selected based on structural characteristics (with or without 1, 14-carbonate moiety) and cytotoxicity *in vitro* for evaluation of the interaction with P-gp in Madin-Darby canine kidney (MDCK)-multidrug resistance-1 (MDR1) and MDCK-wild type (WT) cells (a more P-gp specific model)^13,14^ permeability studies, under two sets of conditions: one with and without a P-gp inhibitor (such as verapamil) to look at whether the test compounds were P-gp substrates or not, one with and without a P-gp substrate (such as digoxin) to look at whether the test compounds were P-gp inhibitors or not. The results demonstrated that the interactions of the taxol analogs T-13 and T-26 with P-gp were reduced. Therefore, the permeability of the Caco-2 cells was enhanced *in vitro*.

The current experiments were performed to study the pharmacokinetic profile and absolute oral bioavailability of T-13, T-26 in S.D. rats after acute intravenous and oral administration at single dose using LC-MS/MS based on highly sensitive and specific analytical methodology. In this paper, the main pharmacokinetic parameters such as elimination half-life (t_1/2_), total area under the curve (AUC_0−∞_), and mean residence time (MRT) were estimated. The studies of pharmacodynamic described herein focused on assessing the oral bioavailability of T-13 and T-26.

## Methods

All methods were carried out in accordance with the EC Directive 86/609/EEC for animal experiments. The study was approved by Ethics Committee for Animal Experimentation of Mudanjiang Normal University (Mudanjiang, China).

### Chemicals

Pure compounds T-13 and T-26 were synthetized and characterized by us as described previously^12^. T-13 or T-26 solutions for injection were prepared in saline immediately before administration. Paclitaxel was provided as a sample from the national institute for the control of pharmaceutical and biological products (NICPBP). Reagents required for LC-MS/MS assays were purchased from Sigma-Aldrich. Tween 80 and ethyl acetate were purchased from Aladdin reagent, Shanghai.

### Characterization of T-13 and T-26

The amount of T-13 and T-26 were determined by using an Agilent 1100 series liquid chromatography system equipped with an Agilent G1313A auto-sampler, a binary pump, a reversed-phase C18 Thermo column (150 mm×2.1 mm, 3 μm) with a precolumn (10 mm×2.1 mm, 3 μm) filled with the same material maintained at 30 °C, and a diode-array detector set at 230 nm. The mobile phase was established using a mixture of acetonitrile and water (70:30, v/v) delivered at a flow rate of 0.2 mL/min, and the injection volume was 20 μL. Detection of T-13 and T-26 were performed using a Thermo Finnigan TSQ Quantum triple quadrupole mass spectrometer equipped with an electrospray ionization (ESI) source (San Jose, CA, USA) in positive ion mode. Optimized mass parameters were as follows: ion spray voltage: 4.0 kV, source temperature: 350 °C, sheath gas (nitrogen) 20 psi, auxiliary gas (nitrogen) 5 psi and collision energy: 17 eV for CA, 19 eV for FA and IFA, 15 eV for IS.

Paclitaxel (retention time 3.07 min) used as internal standard, T-13 (retention time 4.2 min) and T-26 (retention time 5.1 min) stock solutions were refrigerated and calibration curves were designed over the range of 5–10,000 ng/mL (γ^2^ > 0.999). The limit of quantification was calculated to be 5 ng/mL. All data acquired were processed by a computer workstation running Agilent Chemstation Rev.A.09.01 Software. Multiple Reaction Monitoring of T-13 and T-26 and internal standard (PTX) utilized the transitions at m/z 915 → 634, m/z 957 → 901, m/z 876 → 308^15^, respectively.

### Administration of T-13 and T-26 to rats

For the pharmacokinetic study, male S.D. rats (average weight 300 g) were fasted overnight to prevent coprophagia but allowing free access to water. The rats were randomly divided into four groups (6 animals each). The dosages of taxanes used in the pharmacokinetic study were based on the previously published reports^15–17^. For solution, T-13 and T-26 were dissolved in a mixture of Tween 80 and ethanol (50:50, v/v) at 50 mg/mL for intravenous and oral administration, respectively. Group I received the T-13 stock solution diluted with normal saline solution up to 1 mL at a dose of 5 mg/kg for intravenous injection. Group II received T-13 at a dose of 60 mg/kg for oral administration. Group III received the compound T-26 stock solution diluted with normal saline solution up to 1 mL at a dose of 5 mg/kg for intravenous injection. Group IV received T-26 at a dose of 60 mg/kg for oral administration.

Blood samples (~0.20 mL) were collected into heparin coated tubes at 0, 5, 10, 20, 40 min, 1, 2, 4, 6, 8, 12 and 24 h after intravenous injection and 5, 15, 30, 45 min, 1, 2, 4, 6, 8, 12 and 24 h after oral administration. Blood samples were centrifuged at 12,000 rpm for 10 min. The supernatant fractions were transferred into labeled microcentrifuge tubes and stored at −40 °C until LC-MS/MS analysis.

### LC-MS/MS quantification of T-13 and T-26 in plasma samples

The concentration of T-13 and T-26 were determined in plasma by LC/MS as described above. Calibration curves were used for the conversion of the T-13/Paclitaxel chromatographic area to the concentration. Calibrator and quality control samples were prepared by adding appropriate volumes of standard T-13 or T-26 acetonitrile solution to drug-free plasma. Calibration curves were designed over the range 5–10,000 ng/mL (γ^2^ > 0.999). An aliquot (100 μL) of plasma sample was mixed with 100 μL of acetonitrile containing internal standard (PTX, 500 ng/mL), followed by vortex mixing. Then the mixture was added 3 mL of ethyl acetate, followed by vortex gentle agitation for 3 min. The mixture was centrifuged at 4500 rpm for 10 min after liquid-liquid extraction, and then, the organic layer was collected and evaporated until dry. Finally, the residue was reconstituted with 120 μL of a mixture of acetonitrile and water (70:30, v/v) and transferred to a clean vial, followed by centrifugation at 12,000 rpm for 3 min. The supernatant fraction was transferred to auto-sampler vial, and analyzed by HPLC. Under these experimental conditions, the running times of T-13 and T-26 were 6 min and 7 min, respectively.

### Pharmacokinetic data analysis

The pharmacokinetic analysis of concentration-time data obtained after the administration of the compounds T-13 and T-26 were analyzed by a noncompartmental model using Xcalibur^®^ (version 1.3) Software (Thermo Finnigan). The following pharmacokinetic parameters were calculated from the plasma data of Group I and Group III using WinNonlin5.2. Software: total area under the plasma concentration versus time curve, determined by extrapolation from 0 to ∞ after intravenous administration (AUC_iv_), half-life of the terminal phase (t_1/2_) and the mean residence time (MRT). Other parameters such as maximum plasma concentration (C_max_) and the time to reach C_max_ (T_max_) were also analyzed after oral administration in rats.

### Statistical analysis

The data were presented as mean ± standard deviation of at least three experiments. The Mann-Whitney U-test was used to investigate statistical differences at a significance level of P < 0.05. All data processing was performed using the statistical package for social sciences (SPSS) software.

## Results

**Figure 2:**
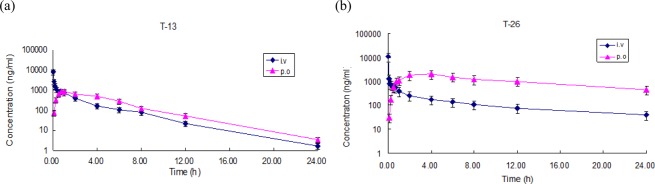
(**a**) Pharmacokinetics of T-13 after intravenous and oral administration to rats. Animals received an intravenous dose of 5 mg/kg and an oral dose of 60 mg/kg. Data are expressed as mean ± S.D., n = 6 at each time point. (**b**) Pharmacokinetics of T-26 after intravenous and oral administration to rats. Animals received an intravenous dose of 5 mg/kg and an oral dose of 60 mg/kg. Data are expressed as mean ± S.D., n = 6 at each time point.

The mean plasma concentration-time profiles and the pharmacokinetic parameters of T-13 and T-26 after intravenous (5 mg/kg) and oral administration (60 mg/kg) in rats were presented in Fig. 2 and Table 1, respectively. As reflected in Table 1, T-13 and T-26 displayed similar values and demonstrated a higher capacity to promote the absorption *in vivo*. Finally, the oral bioavailability of T-13 and T-26 were calculated to be 10.71% and 65.79%, respectively, which were both higher than 5% for paclitaxel^15^.

**Table 1:** Pharmacokinetic parameters after intravenous or oral administration of T-13 and T-26 in rats.

Parameters	T-13		T-26	
	Intravenous (5 mg/kg)	Oral (60 mg/kg)	Intravenous (5 mg/kg)	Oral (60 mg/kg)
t_1/2_ (h)	3.21 ± 0.53	2.97 ± 0.55	10.21 ± 1.72	10.44 ± 1.14
C_max_ (ng/mL)	7990.72 ± 3466.17	921.51 ± 560.87	11017.79 ± 2679.59	2121.12 ± 454.09
T_max_ (h)	—	0.8 ± 0.2	—	3.0 ± 1.1
AUC_0−t_ (ng·h/mL)	3310.69 ± 1333.34	4249.99 ± 2484.31	3439.76 ± 1479.88	25306.31 ± 6148.79
AUC_0−∞_ (ng·h/mL)	3318.42 ± 1333.11	4265.72 ± 2493.71	4054.33 ± 1860.39	32010.17 ± 8537.60
MRT_0-∞_ (h)	2.58 ± 0.87	4.33 ± 0.74	10.08 ± 2.60	15.19 ± 1.71
F (%)	10.71		65.79	

AUC_0−t_: area under the concentration-time curve from time 0 to 24 h; AUC_0−∞_: area under the concentration-time curve from time 0 to _∞_; C_max_: peak concentration; T_max_: time to peak concentration; MRT: mean residence time; t_1/2_: half-life of the terminal phase.

F: absolute oral bioavailability, F = (AUC_p.o_ × Dose_i.v_)/(AUC_i.v_ × Dose_p.o_) × 100%.

## Discussions

This study showed that T-13 and T-26 given oral administration in rats have a bioavailability of approximately 11% and 66%, respectively. The compound T-26 bearing 1, 14-carbonate group was remarkably higher than paclitaxel in rats. In the permeability assay of monolayer Caco-2 cells, compounds T-13 and T-26 showed the highest permeability, with efflux ratios better than ortataxel, an oral taxane used in the phase III clinical trial. T-13 and T-26 were both poor substrates of P-gp, possessed inhibiting effects of P-gp mediated efflux, and their bioavailability was much higher than paclitaxel. The experimental results showed that T-13 improved the oral bioavailability to a limited extent, while T-26 was more effective because it overcomes the effect of P-gp mediated efflux on the one hand, and the dihydroxyl groups at C1 and C14 positions greatly improve its water solubility and increase its transport ability through the blood to the site of effect. The finding further supported the view that P-gp was involved in the low bioavailability of paclitaxel. It was thus clear that simultaneous modifications at the C1, C14 positions introducing water-soluble groups and C7, C10 positions overcoming the interaction with P-gp of paclitaxel can significantly improve its oral bioavailability. In view of its good bioavailability after oral administration, the antitumor activity of T-26 after oral administration was under way.

## Data Availability

All data generated within this study are available from the corresponding author on request.
